# Morally “loaded” labels in the built environment influence perceptions and social judgments

**DOI:** 10.3389/fpsyg.2024.1294220

**Published:** 2024-11-01

**Authors:** Andreas Haga

**Affiliations:** Department of Building Engineering, Energy Systems and Sustainability Science, Faculty of Engineering and Sustainable Development, University of Gavle, Gävle, Sweden

**Keywords:** eco-label, moral, spillover, environment, social judgement, perception

## Abstract

Products and artifacts with morally loaded labels (e.g., environmentally friendly) appear to influence people's perceptions and behaviors. Previous studies have shown that desktop lamps labeled “environmentally friendly” can enhance perceived color discrimination and improve certain reading activities compared to a physically identical lamp labeled “conventional.” This effect may occur because people tend to align their behavior with moral principles. The present study explored the generalizability and robustness of this label effect by asking participants to make trait judgments of photographed faces. In an experimental design, participants evaluated photos illuminated by a desktop lamp that was either labeled environmentally friendly or not labeled at all. The results revealed that participants assigned more positive traits to individuals in the photographs when the lamp was labeled “environmentally friendly,” particularly those with high altruistic values. The pattern was reversed for participants with low altruistic values. Moreover, participants rated the light from the lamp labeled “environmentally friendly” as more comfortable and claimed that the light increased (perceived) visibility. In conclusion, the source of the light—whether from an environmentally friendly or conventional lamp—affects both the evaluation of the light itself and the judgments made about other individuals. This study explores theoretical explanations for these label effects and discusses their potential implications for pro-environmental interventions.

## Introduction

Impression formation is a subconscious process that occurs in our everyday lives, such as when a recruiter interviews a job applicant or when we need to assess whether someone is a friend or a foe (Willis and Todorov, 2006). Evaluating trustworthiness is an essential aspect of social interaction. We do not want to make mistakes when judging others, but whether we like it or not, our judgments are often biased. For instance, Williams and Bargh (2008) showed that holding a warm cup of coffee can bias people toward perceiving others as “warmer” compared to holding a cold cup. This suggests that seemingly irrelevant environmental cues, such as the warmth of a coffee cup, can trigger friendlier social judgments.

The empathy-altruism hypothesis posits that empathic emotions evoke genuine altruistic motivation (Batson and Shaw, 1991). A key research question addressed in the present study is whether “morally” loaded cues can similarly affect social judgments. More specifically, the hypothesis is that an environmentally friendly lamp label may prompt people to make more morally justifiable judgments of other people's traits.

Some product labels are associated with social or environmental responsibility, which can lead morally conscious people to choose organic products over conventional ones and to be willing to pay more money for them (Krystallis and Chryssohoidis, 2005; Sörqvist et al., 2013).

Therefore, “moral” labeling serves as a marketing tool to attract consumers who value social fairness or environmental altruism. In particular, eco-labeling can influence perceptions of products, making them appear superior to conventional alternatives, even when the products are identical (Annett et al., 2008; Haga, 2018; Sörqvist et al., 2015b). This preference bias for products labeled environmentally friendly is known as the *eco-label effect* (Sörqvist et al., 2013, 2015a,b; Haga, 2018).

The magnitude of this effect varies depending on individual differences in environment-related attitudes, reinforcing the hypothesis that the label's influence stems from its appeals to certain individuals' moral consciousness. Altruistic individuals tend to associate higher quality and value with “morally” labeled products and often idealize these options over “conventional alternatives” (Bamberg and Möser, 2007; Schwartz, 1977; Thøgersen, 1999).

People with an altruistic attitude are more likely to engage in pro-environmental behavior, express greater concern for the environment, and often perceive environmentally friendly products as more desirable in various ways compared to conventional ones (Holmgren et al., 2017; Sörqvist et al., 2013, 2015a,b; Yiridoe et al., 2005).

Several studies have shown that eco-labels influence perceptions of the products or objects to which they are attached. Interestingly, these labels can also affect perception and performance in ways that are seemingly unrelated to the labeling itself.

For example, people performed better on proofreading tasks when working at a desk lit by a lamp labeled “environmentally friendly” (Haga, 2018). A similar effect was observed in an experiment on color vision, where participants sorted colored cups more effectively when they believed the desk was illuminated by an “environmentally friendly” lamp, in contrast to when they thought that it was a conventional lamp, even though the two lamps were identical (Sörqvist et al., 2015a).

One possible explanation for this effect is that people perceive differences in production between eco-friendly and conventional products. Tasks such as color sorting and proofreading involve visuospatial processes that depend on good lighting conditions, and the label may enhance performance because people believe the “eco-labeled” lamp is superior to a conventional one.

The current study explores whether eco-labeling can influence subjective ratings (comfort and visibility) and the judgmental dimension that has, arguably, nothing to do with lighting quality, as opposed to proofreading and color vision, and provides an explanation for these effects. For this purpose, the participants were asked to make social judgments of photographed persons, viewed either in the light from a light source labeled “environmentally friendly” or from an unlabeled light source. If the label also has an effect in this context, where the behavior is unrelated to the lamp, it could be because of a strong spillover effect caused by people's belief in the superiority of the lamp, especially for individuals with high environmental concerns.

Since the eco-label appears to enhance visuo-perceptual processes with behavioral consequences, it was hypothesized that participants would report that they could see the stimuli (i.e., photographed faces) more clearly and that they would rate the eco-labeled lamp as more comfortable when the lamp was labeled “environmentally friendly” (H1). Furthermore, behavioral effects of this type of labeling have achieved some generalizability, including performance on a color vision task and proofreading. In this study, the generalizability is tested even further to measure whether personal judgment can be affected by an eco-labeled lamp and if that effect can be predicted by people with high environmental concerns. The hypothesis (H2) is that people will be falsely influenced by the lamp label and, therefore, make more positive judgments of other people in a condition where the moral label is present. The third hypothesis (H3) is that this effect is mainly driven by people who are highly concerned about the environment. If hypothesis three is true, it would not just strengthen the generalizability of the already existing eco-label effect but also show that moral cues, such as labels, probably work like a catalyst to people prone to do morally good and create a spillover effect to domains outside the context at hand.

## Methods

The experimental design was a mixed-participant one. The participants completed the task in both the control condition and the experimental condition. The order of conditions the participants were allocated to do first were counterbalanced. At the end of each task, all participants completed two questionnaires about environmental concerns and value orientation. These questionnaires were then collapsed and divided into a between-group variable.

### Participants

A total of 44 Swedish students (65.3% women) (mean age = 25.04 years, *SD* = 5.48) were recruited to participate in the experiment. All participants were recruited at the University of Gävle and received a small honorarium for their participation. The recruiting process was conducted through an advertisement on a university webpage where students could sign up for the experiment.

### Materials

#### Lamp

A classic incandescent (Osram Classic ECO Superstar) with 30 W input power was used in this study. The lamp had a D efficiency certification and an E14 screw base. The lamp and armature were designed as ordinary light bulbs and ordinary desktop armatures.

#### Statistical tools

Statistical Package for the Social Sciences (SPSS) was used for all analyses in the study.

#### Questionnaire on environmental concern and value orientations

To obtain a highly reliable measure of the three key attitudinal dimensions—biospheric, altruistic, and egoistic orientations—a scale for environmental concern (Stern and Dietz, 1994), along with a slightly different value orientation scale (De Groot and Steg, 2008; Stern et al., 1993), was used and collapsed into one scale. Both measure all three dimensions (biospheric, altruistic, and egoistic). The two altruistic dimensions, the two biospheric dimensions, and the two egoistic dimensions were averaged to obtain three more general indexes of altruistic orientation, biospheric orientation, and egoistic orientation, respectively. The reliability measures of the statistical analysis of Cronbach's α are reported in the Results Section and Table 1.

**Table 1:** Cronbach's alpha for questions measuring environmental concern and value orientation.

	**Cronbach's alpha**
**Questions environmental concern**	
How concerned are you that today's environmental problems will affect …?	
Egoistic	α = 0.91
1. My self	
2. My lifestyle	
3. My health	
4. My future	
Altruistic	α = 0.79
5. All human beings	
6. People close to me	
7. Future generations	
8. My children	
Biospheric	α = 0.87
9. All living things	
10. Plants	
11. Animals	
12. Life at sea	
**Questions value orientation scale**	
How important is each of this for you?	
Egoistic	α = 0.78
1. Social power	
2. Wealth	
3. Authority	
4. Influential	
5. Ambitious	
Altruistic	α = 0.52
6. Equality	
7. A world at peace	
8. Social justice	
9. Helpful	
Biospheric	α = 0.80
10. Preventing pollution	
11. Respecting the earth	
12. Unity with nature	
13. Protecting the environment	
**Environmental concern and value orientation collapsed**	
1. Egoistic	α = 0.87
2. Altruistic	α = 0.79
3. Biospheric	α = 0.86

*N* = 44.

### Design and procedure

A within-participants design was used with the lamp label as the independent variable. In one condition, the lamp was labeled “environmentally friendly,” and in the other, it was labeled “conventional” (although the lamp was identical in both conditions). The lamp label was communicated orally to the participants by the researcher, who told the participants whether the lamp was environmentally friendly or conventional, depending on the lamp's condition. Participants also received information regarding the lamp from a note attached to the lamp (white with black text), which stated that the lamp was environmentally friendly or conventional. Participants first completed the “judgments of lighting conditions and personality traits” task and then filled in the questionnaire on environmental concerns and value orientations. The order between the two lamp conditions in the “judgments of lighting conditions and personality traits” task was counterbalanced between participants, which means all facial pictures were included in both lamp conditions.

Participants were presented with a paper-and-pencil questionnaire and a set of facial photographs. Their task consisted of three phases. In the first phase, they rated how well they could see the picture in front of them (hereafter called “visibility”) on a scale ranging from 1 (not at all well) to 11 (very well). In the next phase, they rated eight personality traits of the photographed person (only one picture from the set). The personality ratings were made on a scale from 1 to 6 and with dichotomous endpoints (i.e., responsible vs. irresponsible; selfish vs. unselfish; not environmentally friendly vs. environmentally friendly; cold vs. warm; dishonest vs. honest; wasteful vs. economic; ruthless vs. charitable; uninterested vs. clever) (Asch, 1946). In the final phase, participants rated how comfortable it had been to work under the lamp's illumination on a scale ranging from 1 (not at all comfortable) to 11 (very comfortable). The three phases were repeated 16 times. Half (eight pictures/cycles) was conducted in one lamp condition, and the other half (eight pictures/cycles) was conducted in the other lamp condition.

An aggregated score for personality judgments was calculated across eight judgmental dimensions and eight photographed individuals in each lamp-label condition (a total of 64 personality judgments per condition). Some of the judgmental dimensions were inverted to ensure that higher values consistently represented a more positive evaluation (e.g., a high level of responsibility) of the photographed individuals' personalities.

The personality judgments were then averaged into a mean score for each participant in both lamp-label conditions.

## Results

### Cronbach's α

This is a reliable measure for the two scales together in all three dimensions where the following results were obtained: egoistic dimension, *M* = 4.55, *SD* = 1.53, Cronbach's α = 0.87, biospheric dimension, *M* = 5.69, *SD* = 1.29, Cronbach's α = 0.86, and altruistic dimensions, *M* = 6.10, *SD* = 1.04, Cronbach's α = 0.79 (Table 1). The three-dimensional indexes were then adjusted (as proposed by Haugh, 1976) by subtracting the grand mean across all three dimensions from the mean of each environmental concern dimension (egoistic, altruistic, and biospheric). The relative scores that result from this adjustment procedure indicate how much higher (or lower) the score is compared with the other two dimensions. For example, a high score on the biospheric dimension indicates that the person who obtained that score had a relatively high score compared to the scores on the other two dimensions. Thus, an absolute altruistic environmental concern score of 6 on a scale of 1–7 can still be low when the indexes are adjusted if that person also obtains a 6 on the other two dimensions.

#### Mixed analysis of variance

These conclusions were supported by a 2 (Altruistic environmental concern/value orientation: high vs. low) × 2 (Label: eco-friendly vs. conventional) mixed analysis of variance with comfort as the dependent variable. The analysis revealed no significant interaction between the two factors *F*_(1, 42)_ = 0.02, *p* = 0.903, $\eta_{\text{p}}^{2}$ = 0.00, but a main effect of lamp label was found, *F*_(1, 42)_ = 11.64, *p* = 0.001, $\eta_{\text{p}}^{2}$ = 0.22.

Difference scores were calculated for judgments of comfort (*M*_diff_ = 0.95, *SD* = 1.82), and the results showed that participants rated the light created by the light from the lamp in the “environmentally friendly” lamp condition as better than that in the “conventional” label condition. The same analysis was conducted for visibility. The analysis revealed no significant interaction between the two factors *F*_(1, 42)_ = 0.54, *p* = 0.468, $\eta_{\text{p}}^{2}$ = 0.01, but a main effect of lamp label was found, *F*_(1, 42)_ = 7.71, *p* = 0.008, $\eta_{\text{p}}^{2}$ = 0.16. The difference scores were calculated for judgments of visibility (*M*_diff_ = 0.52, *SD* = 1.23), and the results showed that participants rated the visibility created by the light from the lamp in the “environmentally friendly” lamp label condition as better than the light in the “conventional” lamp label condition.

However, the eco-label effect (i.e., the difference between the two lamp-label conditions) on personality judgments varied with individual differences in altruistic environmental concern/value orientation (Table 2). Most notably, the positive correlation between personal judgment and altruistic environmental concern/value orientation indicated that the eco-label effect increases with individual differences in altruism.

**Table 2:** Intercorrelations amongst the variables in Experiment 2 (*N* = 44).

**Variable**	**1**.	**2**.	**3**.	**4**.	**5**.
1. Egoistic (adjusted)	-				
2. Altruistic (adjusted)	−0.42^**^	-			
3. Biospheric (adjusted)	−0.83^**^	−0.15	-		
4. Differences in personality judgements	−0.20	0.47^**^	0.08	-	
5. Differences in comfort	−0.10	0.01	0.12	−0.10	-
6. Differences in visibility	−0.11	0.08	0.10	−0.03	0.76^**^

The table shows correlations between three indexes of environmental concern/value orientations and the difference scores between the “environmentally friendly” lamp label condition and the “conventional” lamp label condition across three dependent variables (comfort ratings, visibility, and personality judgments). ^**^p < 0.001.

**Figure 1:**
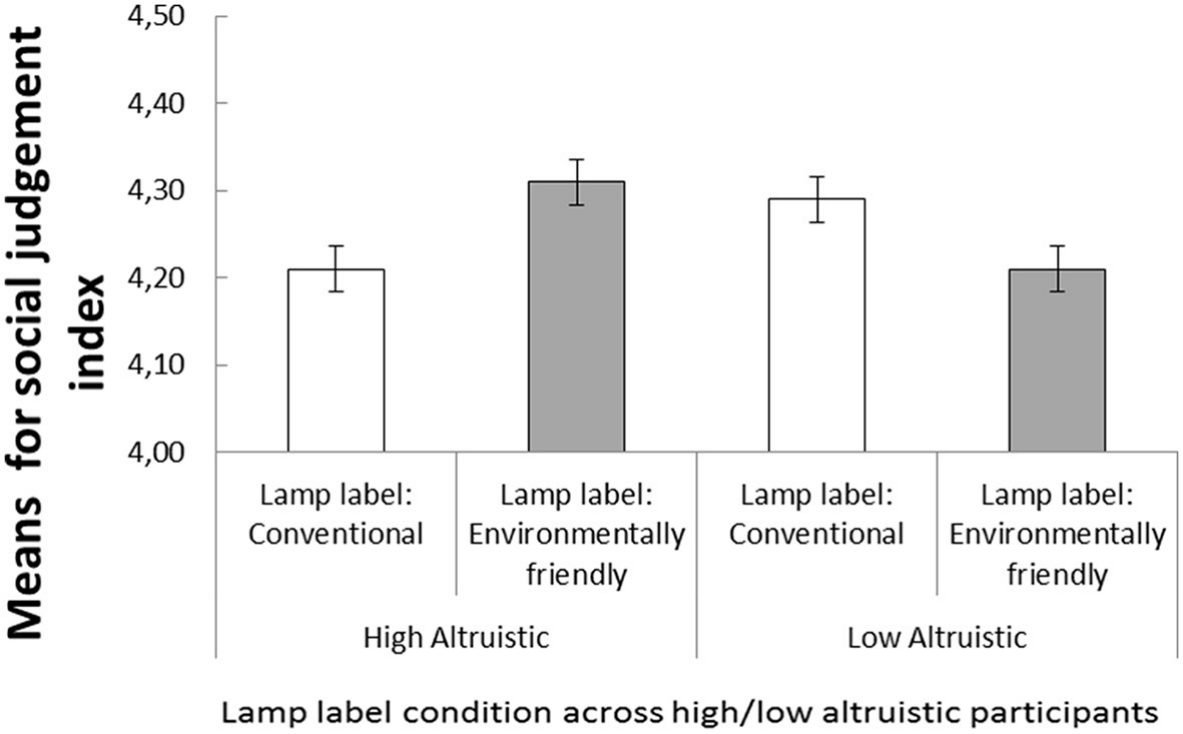
Participants with high altruistic environmental concern/value orientation assigned more positive personality traits to photographed people when the photographs were enlightened by a lamp that was labeled “environmentally friendly” than when the lamp was labeled “conventional.” The opposite was found for participants with low altruistic environmental concern/value orientation. Error bars represent the standard error of the means.

To illustrate the interaction between the lamp label condition and environmental concern/value orientation, the participants were divided into two groups based on a median split (high altruistic environmental concern/value orientation, *M* = 1.93, *SD* = 0.68, vs. low altruistic environmental concern/value orientation; *M* = 0.82, *SD* = 0.44). Figure 1 shows that participants with a high altruistic environmental concern/value orientation evaluated the photographed persons more positively when the lamp was labeled “environmentally friendly,” compared to when the lamp was labeled “conventional,” whereas participants with a low altruistic environmental concern/value orientation evaluated the photographed people less favorably when the lamp was labeled “environmentally friendly” compared to when the lamp was labeled “conventional.” These conclusions were supported by a 2 (altruistic environmental concern/value orientation: high vs. low) × 2 (Label: eco-friendly vs. conventional) mixed analysis of variance with personality judgments as the dependent variable. The analysis revealed a significant interaction between the two factors, *F*_(1, 42)_ = 5.72, *p* = 0.021, $\eta_{\text{p}}^{2}$ = 0.19, while no main effect was significant, *F*_(1, 42)_ =0.13, *p* = 0.721, $\eta_{\text{p}}^{2}$ = 0.003.

The follow-up *t-*test analysis showed that the interaction was driven by differences in altruism levels, with highly altruistic individuals giving more positive judgments in the eco-friendly condition *t*_(42)_ = 2.12, *p* = 0.046, indicating that the eco-label effect was stronger for participants with a higher altruistic environmental concern/value orientation (Figure 1).

## Discussion

Participants assigned more favorable ratings of the light from the light source when it was labeled “environmentally friendly” compared to the lamp labeled conventional. Moreover, the results also showed that the effect of eco-labeling generalizes to subjective evaluations of target objects lit up by the lamp: the participants assigned what are, arguably, more positive personality traits to the photographed persons when the photos were viewed in the spotlight from a lamp with an “environmentally friendly” label. However, this was only true for participants with high altruistic environmental concerns and value orientation.

A likely explanation for the eco-label effect is the strong spillover effect. When this effect extends from pro-environmentally to more pro-social behavior, it suggests that the behavior is driven by deeper, more fundamental predispositions than only favorable perceptions of a product (e.g., a lamp). A more specific explanation of this spillover effect is the halo effect (Thorndike, 1920), where overall impressions and feelings about a person or a brand influence our perceptions of their other qualities. For example, if we believe an eco-friendly labeled lamp is better for the environment, we may also assume it provides better light.

Additionally, the eco-label effect can be understood as a perception bias—people tend to experience and behave according to their expectations (Haga et al., 2016). When exposed to something that aligns with their moral values, individuals act in ways that confirm those values, effectively avoiding inner conflicts (Stern et al., 1985). In this case, altruistic individuals favor the eco-friendly lamp, while those with opposing values may prefer the conventional lamp.

As previously mentioned, the eco-label effect has been demonstrated across a wide range of products and domains, from taste perceptions of food items such as bananas, raisins, and grapes to artifacts such as lamps (Haga, 2018) and even buildings (Holmgren and Sörqvist, 2018). The effect has also been observed in fundamental behaviors like color vision and tasks involving cognitive activities, such as proofreading and language processing. In this study, the eco-label effect was found to influence social judgments, which involve memory and cognitive reasoning. Moreover, the effect appears stronger among individuals who are environmentally conscious and possess altruistic values.

One way to explain this phenomenon is by identifying a single mechanism that underpins all these varied behaviors and perceptions. Alternatively, it may be that multiple mechanisms are at play, depending on the context. For example, when explaining the effect on taste, beliefs about the production process could be a plausible explanation. For color vision and proofreading, the alignment between personal values and external product information (e.g., an eco-label) might encourage deeper engagement with the task. However, these explanations do not fully account for the impact on social judgments.

Why do altruistic individuals rate others more positively when exposed to an eco-label compared to a conventional one? It may be that eco-labels serve as cues that trigger moral responsibility in those with altruistic values, leading to more favorable judgments of others. Research has shown that people rate photographs of others more positively when viewed in an aesthetically pleasing room, judging the person in the photograph as more attractive compared to when the same photographs are viewed in an unattractive room (Maslow and Mintz, 1956). This effect is similar to the one observed in the current study, suggesting that the environment or environmental cues, such as the Thorndike halo effect, can induce positive feelings that extend to other judgments.

It can be argued that pro-social and pro-environmental behaviors may both stem from similar moral obligations (Bamberg and Möser, 2007), supporting the decision to collapse the two scales of value orientation and environmental concern. Several primary studies provide evidence that moral norms contribute to explaining pro-environmental behaviors such as energy conservation (Black et al., 1985), recycling (Guagnano et al., 1995), traveling alternatives (Hunecke et al., 2001), and pro-environmental consumption (Thøgersen, 1999). As reported above, significant studies have found a mean correlation of *r* = 0.33 between a feeling of moral obligation and pro-environmental behavior (Hines et al., 1987). Moreover, the internal attribution of a harmful behavior often triggers emotional guilt reactions (Weiner, 2000). To avoid a mismatch between one's own behavior and social norms, people behave according to their moral obligations (Joireman, 2004).

Comfort and visibility were rated significantly higher in the eco-label condition, while personal trait ratings did not show a significant difference. This may be due to the fundamental differences between perception (comfort and visibility) and behavioral judgments. Moreover, it is probably easier to imagine that increased features such as visibility and comfort are related to lamp type, even for participants with low altruistic values. For more abstract effects such as social judgment, higher altruistic values are necessary to generate motivation for the eco-label effect. Therefore, the likelihood of a spillover effect is greater (Thøgersen and Ölander, 2003).

### Implications and further research

Further research should investigate why eco-labeled products do not appeal to some people. In this study, some valuable insights were found about the eco-labeled effect, which builds on previous research showing that people concerned about the environment are generally more sensitive to the eco-label effect. However, these individuals are not the target group from an applied perspective, as they already think about environmental issues and behave accordingly. The issue is thus that people are low in environmental concern. Research should also explore why some people are not concerned about the environment, find ways to influence such people to behave in favor of the planet, and help deal with climate issues. To make this implication possible, it is necessary to target the underlying mechanisms and barriers to these decisions.

This study did not aim to find solutions to combat climate change but rather to explore the generalizability of the eco-label effect and gain a deeper understanding of human behavior in general. However, an implication for climate change emerging from this study is the potential to design interventions that might induce negative spillover effects, as suggested by the results. Eco-labeling, while useful, may have flaws that could be counterproductive for climate change mitigation efforts and should thus be carefully investigated.

Moreover, the majority of the pro-environmental interventions in the current times, such as promoting collective transport at bus stations and eco-labeling, often mistakenly target people who already care about the environment, resulting in misdirected efforts. For example, people using collective transport at bus stations are already engaging in environmentally friendly behavior, so targeting them leads to poorly allocated resources. Instead, interventions should focus on people with low environmental concerns and be carefully designed to engage them without causing negative spillover or alienating them.

This issue extends to other climate change challenges. For instance, interventions in areas threatened by rising sea levels should be tailored to address those specific risks, while inland interventions should focus on relevant environmental concerns. Future research should prioritize strategies that target individuals who struggle to engage in pro-environmental behaviors. According to Truelove et al. (2014), if promoting pro-environmental behavior leads to positive spillover effects, these investments can offer significant benefits.

## Data Availability

The data that support the findings of this study are available from the University of Gävle but restrictions apply to the availability of these data and so are not publicly available. Data are however available from the authors upon request and with permission of University of Gävle. A request can be made at https://doi.org/10.5878/0q8f-ya14.
